# Tau Is Truncated in Five Regions of the Normal Adult Human Brain

**DOI:** 10.3390/ijms22073521

**Published:** 2021-03-29

**Authors:** Michael G. Friedrich, Amanda Skora, Sarah E. Hancock, Todd W. Mitchell, Paul L. Else, Roger J. W. Truscott

**Affiliations:** 1 Illawarra Health and Medical Research Institute, Wollongong University, Wollongong, NSW 2522, Australia; michaelf@uow.edu.au (M.G.F.); amandagr@uow.edu.au (A.S.); toddm@uow.edu.au (T.W.M.); 2 School of Medicine, University of Wollongong, Wollongong, NSW 2522, Australia; pelse@uow.edu.au; 3 Molecular Horizons, University of Wollongong, Wollongong, NSW 2522, Australia; 4 School of Medical Sciences, University of New South Wales, Sydney, NSW 2052, Australia; sarah.hancock@unsw.edu.au

**Keywords:** Tau, ageing, Alzheimer disease, truncation, protein unfolding, protein truncation

## Abstract

The truncation of Tau is thought to be important in promoting aggregation, with this feature characterising the pathology of dementias such as Alzheimer disease. Antibodies to the C-terminal and N-terminal regions of Tau were employed to examine Tau cleavage in five human brain regions: the entorhinal cortex, prefrontal cortex, motor cortex, hippocampus, and cerebellum. These were obtained from normal subjects ranging in age from 18 to 104 years. Tau fragments of approximately 40 kDa and 45 kDa with an intact N-terminus retained were found in soluble and insoluble brain fractions. In addition, smaller C-terminal Tau fragments ranging in mass from 17 kDa to 25 kDa were also detected. These findings are consistent with significant Tau cleavage taking place in brain regions from 18 years onwards. It appears that site-specific cleavage of Tau is widespread in the normal human brain, and that large Tau fragments that contain the N-terminus, as well as shorter C-terminal Tau fragments, are present in brain cells across the age range.

## 1. Introduction

Alzheimer’s disease (AD) is the most common form of dementia; however, the reasons for the development of AD are poorly understood. The disease is characterised by the accumulation of large misfolded aggregates of two proteins: β-amyloid, derived from amyloid precursor protein (APP), which forms extracellular plaques; and Tau, which polymerises into intracellular neurofibrillary tangles (NFTs). Tau aggregation appears to correlate best with the progression of AD [1,2] and it has been proposed that the neurotoxicity of β-amyloid is mediated predominantly via Tau [3]. Tau has also been linked to several other neurodegenerative disorders, including supranuclear palsy [4], and frontotemporal dementia with Parkinsonism [5]. It is not known what triggers Tau, a soluble unstructured protein, to form paired helical filaments (PHFs) and other potentially toxic intermediates.

Tau in the brain is comprised of six isoforms ranging in size from 36.7 to 45.9 kDa and can be categorised by the number of repeat binding domains within the C-terminus. Within normal neurons, positively charged domains within the C-terminus of Tau bind to negatively charged microtubules. It is thought that formation of abnormal aggregates and PHFs in AD is modulated by posttranslational modifications (PTMs), in particular, the truncation of the C-terminal binding domain and phosphorylation [6]. Protein truncation is well-established to lead to protein unfolding and aggregation [7] and it is linked to an altered conformational state of Tau [8]. Whilst these modifications have been linked to the toxicity of Tau, additional modifications have been found [9,10,11,12,13,14,15].

A number of sites of Tau truncation appear to be associated with AD, including cleavage at Asp 421 and Glu 391 [16,17] and are summarised in a recent review [18]. Proteolytic fragments of Tau are prone to aggregate and also initiate apoptosis [19,20,21,22]. Isolated PHFs from AD patients contain Tau truncated at Glu 391 [23]; the components of PHF have been visualised by cryo-EM [24]. Current evidence also suggests a role for C-terminally truncated forms of Tau spreading within the brain and causing neuronal death [25]. Some researchers have proposed a therapeutic approach to suppress Tau pathology in AD by inhibiting Tau truncation [26,27]. Despite a large body of information on Tau truncation in AD [18], little is known about truncation in normal brain tissues. 

Evidence is accumulating that Tau is a long-lived protein (LLP) and therefore over time may be subject to the range of PTMs for example racemisation [10,28] and truncation [29,30,31] that characterise this group of proteins. In order to examine whether a significant truncation of Tau could be detected in normal human brains, specific regions were examined across the age range.

Previous Tau studies have focused mostly on one brain region. In the present survey, we sought to provide some baseline information that could act as a platform for future studies to examine the role of Tau truncation in AD. Does significant Tau truncation exist in the normal human brain? Is there much variation in pattern, or are the same or similar Tau fragments present in all individuals? If truncation is found, at what age can it be detected and is it localised in particular brain regions? 

## 2. Results

**Figure 1:**
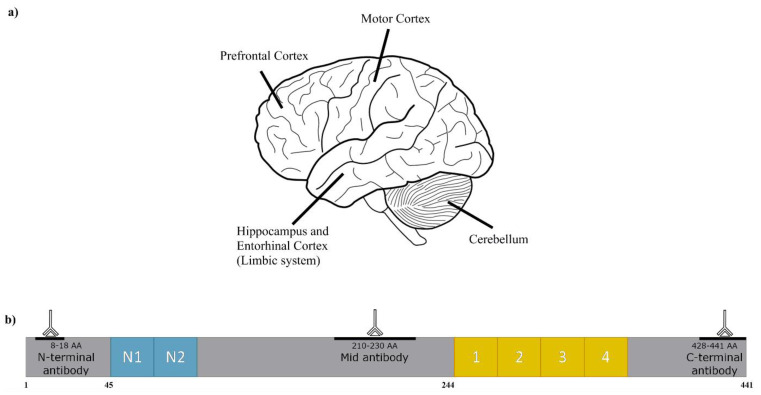
Schematic of the brain regions examined, and the antibodies used to probe Tau. (**a**) Diagrammatic representation of the human brain with the regions highlighted. As illustrated, both the hippocampus and entorhinal cortex are internal. (**b**) Schematic of the Tau 441 isoform with highlighted antibody epitope recognition sites.

In order to gauge if truncation of Tau could be detected in human brains, three different Tau antibodies were used to target epitopes in the middle, the N-terminus, or the C-terminus of the six Tau isoforms present in the human brain (Figure 1). These antibodies have been used in a number of other Tau investigations, e.g., [32,33,34,35,36].

Significant regional and solubility differences in Tau were found with some person-to-person variation in the Tau profiles, as expected for human samples. In all cases, the amount of Tau in the soluble fraction was significantly lower than in the insoluble fraction (~3 to 10-fold less depending on the particular brain region). This is in agreement with the literature where cellular Tau is bound primarily to insoluble microtubule filaments [37]. In agreement with other studies, lower levels of Tau in both the soluble and insoluble fractions were found in the cerebellum [38,39]. 

In order to visualise the distribution of immunoreactive species, samples were first analysed using an antibody directed to the central portion of Tau (see Figure 1). This mid-sequence antibody (ab80579) shows weak non-specific reactions [40]. Selected samples across the age range were then also analysed with the two terminal antibodies (Figure 1). The results of the Western blots using these three antibodies are discussed separately below. 

### 2.1. Mid-Sequence Antibody

**Figure 2:**
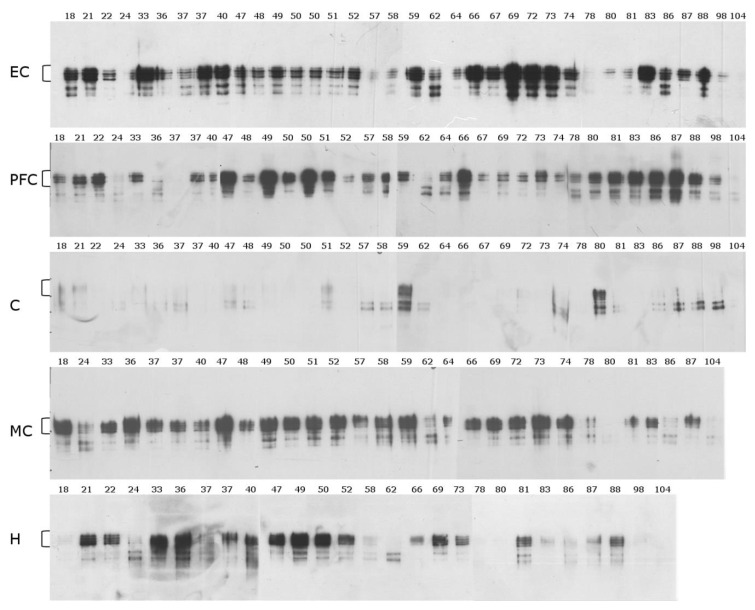
Insoluble Tau using a mid-sequence antibody. Western blot analysis using an antibody raised to an epitope in the middle of Tau. Insoluble Tau was probed with an antibody specific to the mid-sequence of Tau (see Figure 1) in five brain regions: the entorhinal cortex (EC), prefrontal cortex (PFC), cerebellum (C), motor cortex (MC), and hippocampus (H). The ages are displayed above each lane. Brackets correspond to the migration zone of the full sequence Tau isoforms.

**Insoluble fraction.** The mid-sequence antibody typically gave rise to bands of a molecular weight corresponding to four of the six known Tau isoforms (Figure 2). Since all six isoforms of Tau were detected when a commercial Tau protein ladder was employed with this antibody, these data suggest that the lack of detection of two Tau isoforms in human samples was due to the modification of some epitopes [40], or that two Tau isoforms are present at much lower levels in the human brain than the other four. This pattern was similar in all brain regions examined (PFC, H, MC, EC), with the exception of the cerebellum, which displayed fewer bands than the other brain regions. The cerebellum also showed an apparent age-dependent trend with two lower molecular weight (MW) immunoreactive Tau bands at ~45 kDa and 40 kDa dominating, particularly from age 80 onwards (Figure 2). These two bands were also present in some other brain regions. 

Although brain Tau patterns were generally reproducible across the age range, there were some notable exceptions. For example, the 24-, 57-, 58-, 78-, and 104-year-old samples showed an almost complete absence of Tau immunoreactivity (Figure 2). This finding could be due to the lower-than-expected brain pH of these individuals (see Supplementary Materials Figure S1)

**Figure 3:**
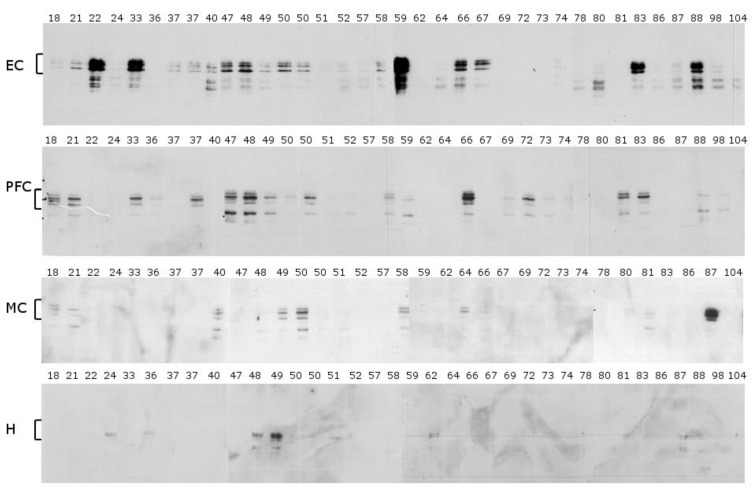
Soluble Tau using a mid-sequence antibody. Western blot analysis using an antibody raised to an epitope in the middle of Tau. The soluble fraction was probed with an antibody specific to the mid-sequence of Tau (Figure 1) in five brain regions: the entorhinal cortex (EC), prefrontal (PFC), motor cortex (MC), and hippocampus (H). The ages are displayed above each lane. Brackets correspond to the migration zone of the full sequence Tau isoforms. The cerebellum is not displayed due to the lack of immunoreactivity in this region.

**Soluble fraction.** The corresponding soluble fraction from each of the brain samples differed more than the insoluble fraction both in terms of inter-donor and regional variability (Figure 3). Overall, the greatest amount of soluble Tau was detected in the EC followed by the PFC, although there was considerable person-to-person variation. One consistent finding was that the soluble fraction contained fewer immunoreactive Tau components that also migrated as tighter bands in comparison to the Tau in the corresponding insoluble samples from the same donor. This was most clearly evident in the PFC samples. In most cases, only one or two major bands were observed. No soluble Tau was detected in any sample from the cerebellum. 

### 2.2. N-Terminal Antibody

A subgroup of the same brain samples was also probed with antibodies to the N- and C-terminus of Tau to search for fragments of Tau that may not have been detected with the mid-sequence antibody. This lack of immunoreactivity has been observed if significant PTMs are present within the antigen [30,40]. In this instance, age-related PTMs of the central part of Tau may disrupt antigen recognition. 

**Figure 4:**
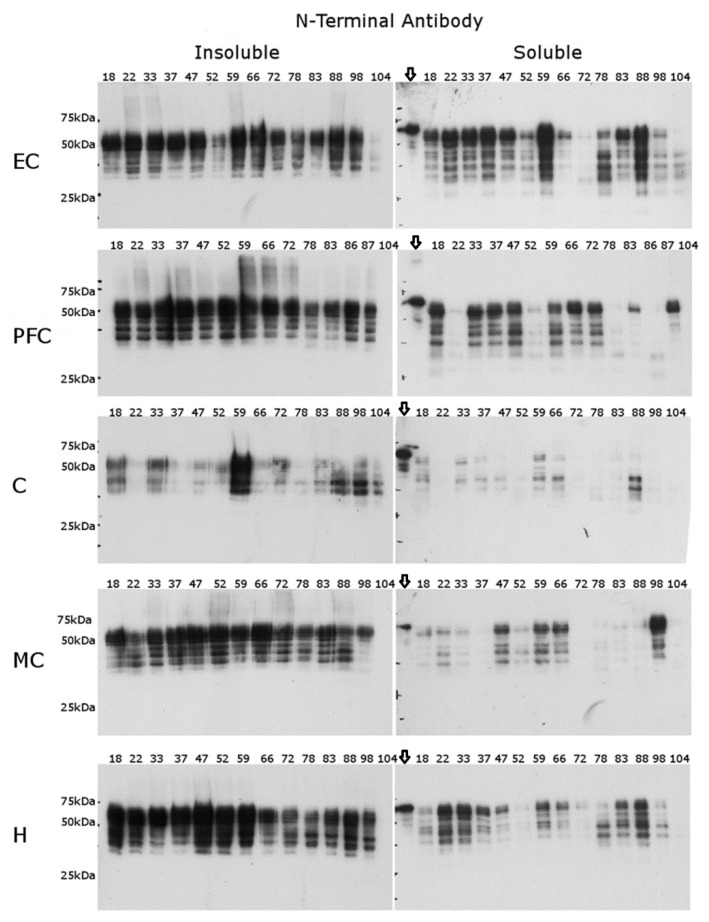
Soluble and insoluble fractions from the same brain regions using an N-terminal antibody. Western blot analysis of the N-terminus of Tau. Both insoluble and soluble fractions were probed with an antibody specific to the N-terminus of Tau in five brain regions: the entorhinal cortex (EC), prefrontal cortex (PFC), cerebellum (C), motor cortex (MC), and hippocampus (H). The ages are displayed above each lane. Downward arrow Tau 441 standard.

The N-terminal antibody blots were generally more reproducible in pattern between the donors across the age range (Figure 4). The N-terminal antibody also allowed for a better visualisation of low-molecular-weight (LMW) Tau fragments (below 50 kDa) that were evident at an early age (Figure 4). These Tau fragments ranged in apparent mass from 30 to 37 kDa in the insoluble fraction, and from 25 to 37 kDa in the soluble fraction. Most fragments appeared to be of the same size, based on mobility, in the insoluble and soluble brain fractions, although a greater number of truncated Tau species were present in the soluble fraction. This was particularly noticeable in the cortical brain regions where several low MW bands (~25 kDa and 28 kDa) were observed.

### 2.3. C-Terminal Antibody

Since truncation appeared to be a major modification of Tau as judged by the N-terminal antibody blots, the same brain extracts were investigated using an antibody raised to the last 17 amino acids of Tau. One rationale for this approach was that if a single cleavage site, or a few localised sites, were involved in the truncation of Tau, then fragments retaining the C-terminus may be observed. In addition, since they would be relatively small, they may be more likely to be present in the soluble fraction. 

**Figure 5:**
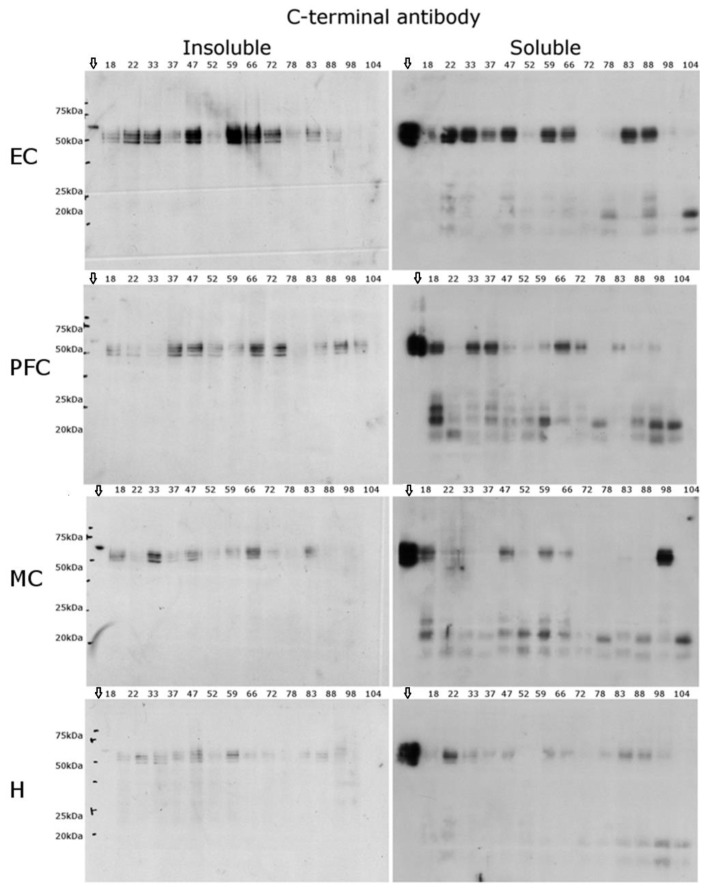
Soluble and insoluble fractions from the same brain regions using a C-terminal Tau antibody. Western blot analysis of the C-terminus of Tau. Both insoluble and soluble fractions were probed with an antibody specific to the C-terminus of Tau in four brain regions: the entorhinal cortex (EC), prefrontal cortex (PFC), motor cortex (MC), and hippocampus (H). The ages are displayed above each lane. Downward arrow Tau 6 ladder standard loaded onto each gel. The cerebellum is not shown due to the lack of immunoreactivity in this region.

As seen in Figure 5, the overall immunoreactivity for the insoluble fractions with the C-terminal antibody was reduced by comparison with the N-terminal antibody (Figure 4). In the EC, an apparent age-dependent loss of full sequence Tau was apparent, such that by age 60 to 70, little to no immunoreactive Tau was observed in the insoluble fraction. A possible explanation for this is that the majority of Tau in older people has been modified by truncation at the C-terminus. Since the samples from younger individuals showed similar immunoreactivity using the N-terminal antibody and C-terminal antibody (Figure 4 and Figure 5). In the absence of other data, however, it is not possible to rule out that PTMs at the C-terminus could explain these findings. A similar but less distinct age-related phenomenon was observed in the other brain regions.

The blots of the soluble fractions were instructive. In the cortical regions of the brain, several LMW immunoreactive bands were observed (Figure 5), and these were particularly evident in the PFC and MC. These Tau fragments were present in samples across the age range. In some donors, four LMW immunoreactive bands were detected of varying intensities, but two bands—one at ~20 kDa and one at ~17 kDa—were consistently present, particularly in the PFC and MC. These same LMW immunoreactive bands were also detected by Western blotting using another C-terminal antibody (DAKO, A0024). While exact sites of cleavage were not determined, it is of note that some fragments migrated to regions that matched known sites of truncation such as Asn 255 [25]. 

Whilst Tau was detected in the PFC, EC, MC, and H using the C-terminal antibody, no Tau was detected in the cerebellum. To confirm if this was due to an antibody and/or concentration issue, both antibody and sample concentrations were increased (five-fold); however, no Tau was observed, suggesting that little Tau with an unmodified C-terminus is present in this region. 

One of the most widely used Tau antibodies for Western blotting is the Dako antibody, which recognises the four microtubule binding regions in the C-terminal portion of Tau [41]. We employed this antibody for one of the brain regions (PFC) to show that the overall amount of Tau immunoreactivity in the extracts from the individuals was similar (Figure S2). As shown in Figure S2b, the overall staining intensity for each lane was similar, the only exception being the 104-year-old individual who displayed almost no Tau immunoreactivity with the other three Tau antibodies.

The Dako antibody confirmed that for individuals above the age of 70 years, a common feature was immunoreactivity being associated with smaller Tau fragments (Figure S2a). Dot blot analysis (Figure S3) showed that the amount of overall Tau immunoreactivity across the age range did not differ markedly, although this information cannot be considered as strictly quantitative.

In summary, the insoluble fractions displayed a similar degree of Tau truncation between brain regions when probed with N-terminal and mid-region antibodies. By contrast, little or no truncation was detected with the C-terminal antibody. This finding suggests that the truncation of most Tau (i.e., insoluble) with age takes place primarily from the C-terminus. Overall, it would appear that truncation is an abundant modification in at least four of the human brain regions studied.

### 2.4. Limitations of the Study

The results of this study need to be interpreted in the light of the following caveats. Although the three antibodies that were used have been employed in many other published experiments on Tau, e.g., [32,33,34,35,36], we did not independently validate them. Since this was not intended to be a quantitative study, loading controls were not incorporated. Instead, human brain regions were carefully dissected, and care was taken to ensure that all procedures were carried out reproducibly. Lastly, this survey was set up largely to determine if Tau truncation could be detected in separate brain regions in normal controls and to determine if the fragmentation pattern was consistent across the age range. In future investigations, this information will act as a basis for studies on the same brain regions of AD patients.

## 3. Discussion

In this study, significant truncation of Tau has been demonstrated in the normal human brain. Extensive truncation of Tau was observed in the EC, PFC, MC, and H, even at age 18 years. Furthermore, truncation of Tau in the different brain regions appears to be site-specific with the N- and C-terminal portions of Tau being retained in the cell. Similar results have been reported recently in the aged human brain following the administration of ^13^C_6_leucine [42]. This phenomenon was most evident in the cortical brain regions with two prominent immunoreactive C-terminal peptides being present in all MC and PFC samples. These ~17 kDa and 20 kDa fragments were soluble, implying that they were not proteolysed further and that they may interact more weakly with microtubules. 

If Tau is a long-lived protein as recent data suggests [43], then it may not be surprising that it undergoes cleavage over time. As with other proteins such as crystallins [29,30,44], significant cleavage can be measured even in samples from people in the second decade of life. 

Although masses as determined solely based on SDS PAGE mobility must be treated with caution, since it is known that each of these polypeptides retains the C-terminus, it is possible to calculate approximate regions of proteolysis. For the larger (20 kDa) fragment, peptide bond cleavage in the region of residues S241 to G261 would be consistent with this observation and, using a similar method of analysis, peptide bond cleavage in the region of residues G271 to K290 would account for the 17 kDa Tau fragment. One possible candidate for the 20 kDa Tau fragment is cleavage between N255 and V256, which has been observed in mouse models [25], and this cleavage also occurs spontaneously in Tau when it is incubated for extended times [15]. As discussed below, such Tau cleavages could occur due to protease activity or spontaneous cleavage at Ser [45], and Asn [29] may be responsible.

If one peptide bond is cleaved, then the remaining (i.e., N-terminal) portion should also be observed using the N-terminal antibody. Matching the corresponding portions of Tau is complicated by the fact that intact Tau species of different masses are present in the brain [46] and additional truncations could occur. Nonetheless, fragments of Tau on SDS PAGE were present in brain regions across the age range, as detected by the N-terminal antibody (Figure 3), and these fragments were calculated to be ~15 kDa to 20 kDa lower in mass than the longest intact canonical Tau isoform present in the human central nervous system. Other less prominent immunoreactive bands using the C-terminal antibody were also observed in some brain regions, in particular the PFC (Figure 4), suggesting that other sites of proteolysis may also be evident. 

Whilst truncations have been ascribed to proteolytic cleavage by caspases and calpains [18,47], it should be noted that many Tau cleavage sites are not well-characterised, and in some cases do not match the substrate cleavage sites of any protease. One intriguing possibility is that the spontaneous non-enzymatic cleavage of Tau [44,48] could account for the cleavage seen. Previous research has shown that, following prolonged incubation, Tau cleaves on the carboxyl side of asparagine [15]. Cleavage on the C-terminal side of Asn is a recognised spontaneous event [29,44]. Such results may be expected if Tau in the human brain is an LLP [9,49]. Several sites of isoAspartate formation in Tau have been reported [50], which supports the identification of Tau as an LLP. It has only been appreciated recently that there are numerous proteins in the human body that turn over slowly, some of which do not turnover at all during the human lifespan [51]. Over time, LLPs accumulate PTMs that compromise their normal conformation and function. Extensive non-enzymatic PTMs include racemisation [10,52], deamidation [10,53], and truncation [30,54]. These spontaneous processes appear to be localised to unstructured regions of proteins [55]. 

This study shows that Tau truncation is widespread in the human brain and is present early in life. It appears to occur primarily in the C-terminal region, suggesting a common mechanism of truncation. Whilst the Tau fragments did not differ greatly between regions, significant differences were observed in the amount of immunoreactive Tau. In particular, the cerebellum was found to be quite different from the other brain regions investigated, having a lower Tau content. This is in agreement with previous studies [38,39]. Although truncation of Tau was a feature common to all brain regions, there were differences in the amount of truncation observed. There was limited evidence in this study to show any definite age-related trends. 

If Tau is indeed an LLP, then its truncation would not be surprising. This can occur via enzymatic [56] or spontaneous processes. Over time, spontaneous cleavages, in particular at Asp [44], Asn [29], and Ser [45] residues, take place. As to why Tau fragments appear not to be degraded further within neurons (or possibly glial cells), it can be speculated that they remain tightly bound to microtubules or that other common age-related PTMs such as isomerisation and racemisation may impair the ability of the cell’s protein degradation machinery to adequately process these modified polypeptides [57]. In other human tissues, significant cleavage as well as other PTMs can be found in life-long proteins isolated from people even as early as the second decade of life [58].

The effect of Tau truncation on normal neuronal function is not understood; however, the toxicity of certain C-terminally truncated Tau species and their relationship to AD has been documented [59,60,61,62]. For example, a recent study documented an increase in the content of soluble Tau fragments truncated at Asp 314 in cognitive disorders, such as AD [63]. In particular, a significant body of research has highlighted the aggregative and toxic properties of Tau truncated at Asp 421 and Glu 391 [19,60,64,65,66]. A diverse range of interactions has been found for these cleaved Tau polypeptides; for example, Tau truncated at Asp 421 accumulates within mitochondria. [67]. Similar mitochondrial accumulation has been found for N-terminally truncated Tau [68] leading to synaptic deterioration associated with AD. Tau truncated at 391 is toxic and self aggregates [69,70]. Truncated Tau has also been shown to interact with the plasma membrane and to alter the blood–brain barrier [71]. Additionally, Tau fragments arising from proteolysis act as nucleation sites and promote Tau aggregation [21]. These various studies on the toxicity of truncated Tau species may need to be interpreted in the context that Tau truncation appears to be quite widespread in normal human brains. 

It is possible that the exact sites of Tau cleavage are crucial for the biological activity of the resulting fragments. If this is true, then it is important in future to thoroughly characterise the cleavage sites of Tau in normal human brains and in brains from those people affected by AD and other diseases associated with the aggregation of Tau. 

## 4. Materials and Methods 

### 4.1. Homogenisation of Human Brain Tissue

Frozen grey matter from five regions (the entorhinal cortex (EC), hippocampus (H), dorsolateral prefrontal cortex (PFC), motor cortex (MC), and cerebellum) from neurologically normal human brains were obtained from the NSW brain bank with ethics approval from the University of Wollongong Human Ethics Committee (Ethics #11/267). Identification and sectioning of each brain region was conducted by the NSW brain bank. Brain tissue was kept at −80 °C until homogenisation. After homogenization, soluble and insoluble fractions were divided into aliquots (to limit freeze–thaw cycles) and stored at −80 °C until analysed. The age range of the brains examined was 18 to 104 years (*n* = 36) (see Table S1). The sample homogenisation was carried out as previously described [72]. Briefly, approximately 100 mg of frozen brain tissue was homogenised in 1.5 mL of 20 mM Tris buffer pH 7.4, containing 2 mM EDTA, 20 mM dithiothreitol, and protease inhibitor cocktail (P8340, Sigma Sigma Aldrich, St Louis, MO, USA) using a bead homogeniser (FastPrep-24, MB Biomedicals, Thermo Scientific, Rockford, IL, USA) set to speed 6 for 40 s as per the manufacturer’s instructions. To minimise the disruption of Tau interactions, no detergent was added to the homogenisation buffer. After homogenization, each sample was immediately transferred to a glass tube (5 mL) and centrifuged at 1000× *g* for 10 min at 4 °C. The resulting pellet was considered to be the insoluble fraction. The supernatant was further centrifuged at 10,000× *g* at 4 °C for 10 min and 100,000× *g* at 4 °C for 30 min for the isolation of mitochondrial and microsomal pellets, respectively. The remaining supernatant is referred to as the soluble fraction. The protein concentrations of both soluble and insoluble fractions were determined using a BCA assay (Thermo Scientific, Thermo Scientific, Rockford, IL, USA).

### 4.2. Western Blots

Protein samples from both the soluble and insoluble fractions (10 µg) were separated using 12% SDS PAGE. The proteins were transferred onto polyvinylidene difluoride membranes (PDVF) (Thermo Scientific) using a Transblot Turbo^TM^ Transfer System (BioRad, Hercules, CA, USA). The PVDF membrane was blocked with 2.5% *w/v* skim milk powder (Oxoid, Thebarton, SA, USA) for 1 h and incubated with one of three different antibodies raised against either the middle Sequence (Abcam, ab80579 dilution 1:4000), C-terminus (Santa Cruz Biotechnology, Santa Cruz, CA, USA, sc-1995 dilution 1:1000), and/or the N-terminus (Abcam, Cambridge, MA, USA, ab74137 dilution 1:5000) of Tau for 16 h at 4 °C. Secondary antibodies were added at a dilution of 1:4000 (donkey anti-goat IgG-HRP, Santa Cruz, sc-2020; and goat anti-mouse IgG-HRP, Abcam, ab6789) for 2 h at 22 °C. The immunoreactive proteins were visualised using enhanced chemiluminescence exposed on Amersham ^TM^Hyperfilm (GE Healthcare). Additionally, selected samples were probed by western blot with the Dako Tau antibody (DAKO, Santa clara, CA, USA, A0024 dilution 1:10,000, secondary, abcam, 97051, 1:10,000. A Tau protein ladder containing the six Tau isoforms (Sigma T7951, Sigma Aldrich, St. Louis, MO, USA) and Tau 441 (rPeptide T1001-1, Athens, MO, USA) were used as standards. Chemiluminescence images were saved as TIFF files.

Samples were examined without loading controls. Age is known to affect the amount of a number of brain proteins [73] and we assessed protein loading using duplicate SDS-PAGE gels stained with Coomassie blue. 

## Supplementary Materials

The following are available online at https://www.mdpi.com/article/10.3390/ijms22073521/s1, Supplementary Figure S1: pH of brain tissue as a function of post-mortem interval (PMI). Samples with little, or no, Tau (Triangle) as detected by the Mid-sequence antibody; Supplementary Figure S2: Western blot of the (a) soluble and (b) insoluble PFC fractions; Supplementary Figure S3: Estimation of Tau in the soluble fractions of the PFC; Table S1: Complete demographics of brain donors (including cause of death) from which tissue was ob-tained from the dorsolateral prefrontal cortex.

## Abbreviations

EC	entorhinal cortex
PFC	prefrontal cortex
H	hippocampus
MC	motor cortex
C	cerebellum
