# Supplementary material for: Tau Is Truncated in Five Regions of the Normal Adult Human Brain
Source: Int J Mol Sci. 2021 Mar 29;22(7):3521. doi: 10.3390/ijms22073521 (PMC8036332; doi:10.3390/ijms22073521)
Supplement: Supplementary file 1 [file ijms-22-03521-s001.pdf]

Table S1. Complete demographics of brain donors (including cause of death) from which tissue was obtained from the dorsolateral prefrontal cortex.

Age (Years)	Sex	PMI (hours)	Brain pH	Cause of death - category	Cause of death - clinical
18	Male	28.5	6.7	Cardiac	Primary cardiac arrhythmia
21	Female	39.5	6.83	Cardiac	Primary cardiac arrhythmia
22	Male	50	6.86	Trauma	Blunt trauma
24	Male	43	6.27	Cardiac*	Undetermined (but consistent with idiopathic cardiac arrhythmia).
33	Female	24	6.77	Cardiac	Cardiac arrhythmia; myocardial fibrosis
36	Male	34	6.67	Cardiac	Acute chronic cardiac failure
37	Male	14.5	6.46	Cardiac	Presumed Cardiac Dysrhythmia due to natural cause
37	Male	24	6.7		Unascertained.
40	Male	27	6.79	Vascular	1.a) Pulmonary thromboemboli b) deep venous thrombosis
47	Male	27	6.66	Cardiac	Ischaemic heart disease.
48	Male	17	6.62	Cardiac	Ischaemic heart disease. Coronary artery atheroma
49	Male	38	6.92	Cardiac	Coronary Artery Disease
50	Male	40	6.87	Cardiac	Haemopericardium
50	Male	34	6.77	Cardiac	Acute Myocardial infarction
51	Male	35	7	Cardiac	cardiomegaly
52	Male	36	6.82		Undetermined.
57	Male	18	6.39	Cardiac	Myocardial Infarction
58	Male	39	6.49	Cardiac	Ischaemic heart disease.
59	Male	15	6.54	Cardiac	Hypertensive and atherosclerotic heart disease
62	Female	35	6.06	Cardiac	Hypertensive and atherosclerotic heart disease
64	Male	17	6.55	Cardiac	Haemopericardium
66	Male	32	6.66	Cardiac	cardiomegaly
67	Male	25	6.7	Cardiac	Hypertensive Heart disease
69	Female	39	6.72	Cardiac	Coronary Artery Disease and asthma
72	Female	25	7	Cardiac	Atherosclerotic cardiovascular disease
73	Female	45	6.86	Cardiac*	Atherosclerotic cardiovascular disease.
74	Female	20	6.59	Cancer	Cancer of breast, liver and bone metastases
78	Female	45	6.05	Toxicity	Multiple drug toxicity (7-amino nitrazepam, nitrazepam and dextropropoxyphene)
80	Male	12	6.5	Respiratory	Emphysema
81	Male	29	6.57	Cardiac	Heart Failure
83	Male	10	6.67	Respiratory	Pulmonary embolus
86	Female	14.5	6.36	Infection	Septicaemia; gangrenous foot; peripheral vascular disease
87	Female	5	6.38	Cancer	Metastatic breast cancer
88	Male	9	6.36	Respiratory	Pneumonia; Debility; Chronic obstructive airways disease
98	Female	6	6.7	Respiratory	Pneumonia; congestive cardiac failure
104	Female	27	5.89	Respiratory	Bilateral bronchopneumonia

* Donor was taking a statin (Lipitor) at time of death.

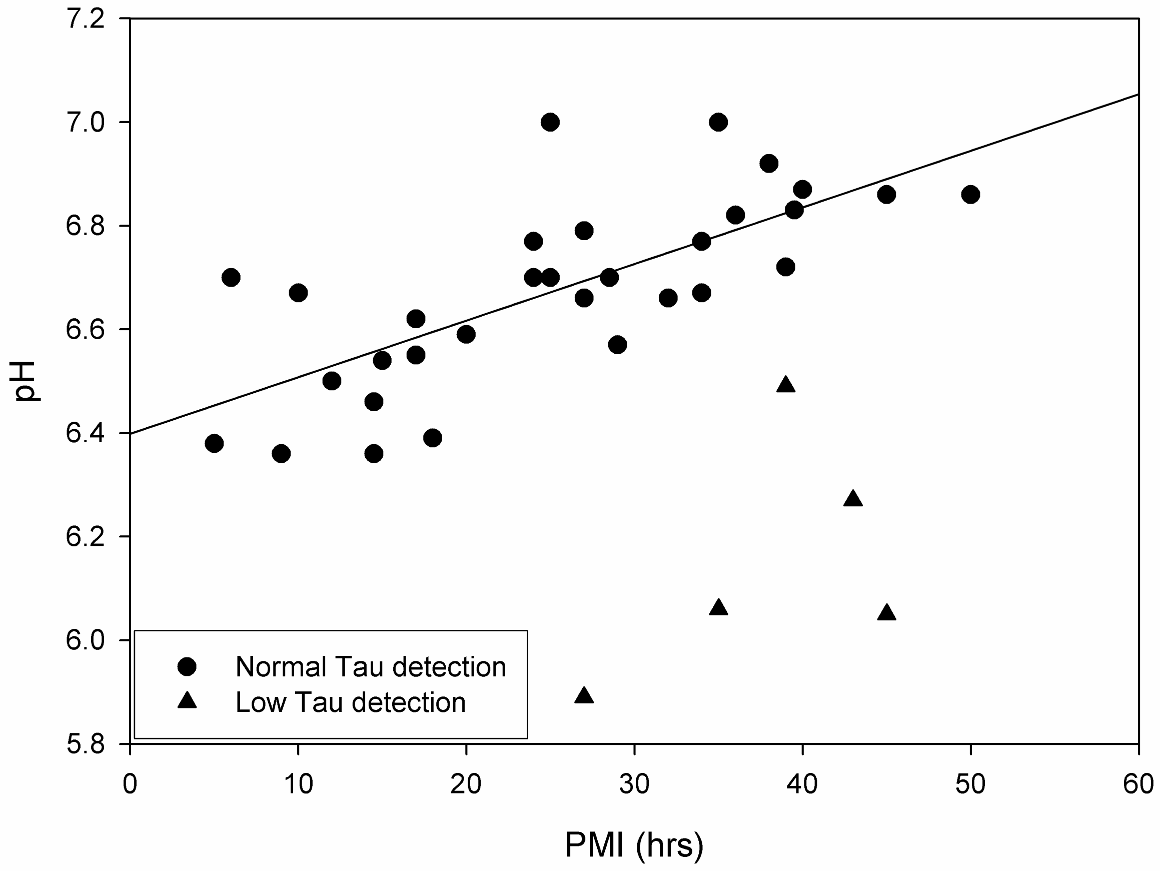

Supplementary Figure S1. pH of brain tissue as a function of post-mortem interval (PMI). Samples with little, or no, Tau (Triangle) as detected by the Mid-sequence antibody.

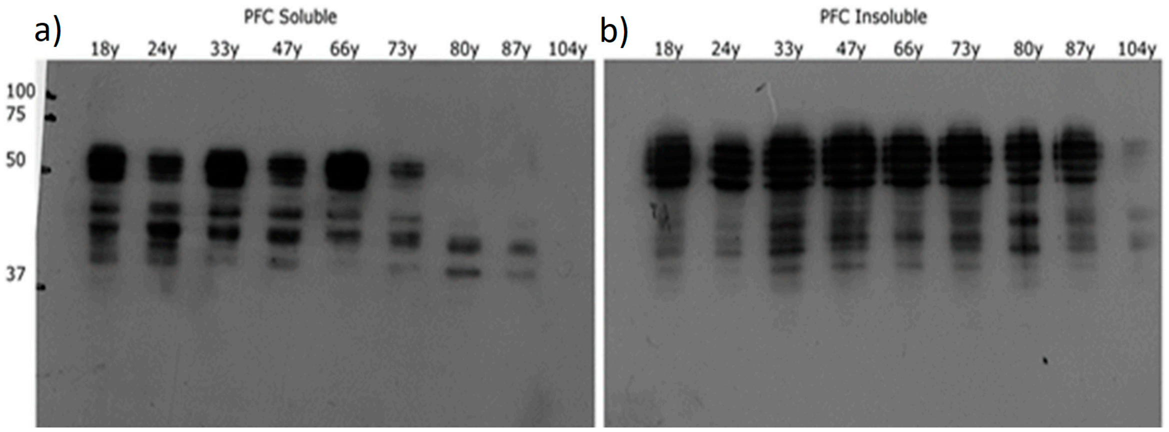

Supplementary Figure S2. Western blot of the a) soluble and b) insoluble PFC fractions. 10µg of protein was loaded per well as determined by BCA assay. Both fractions were probed by the with the Dako Tau antibody specific to the C-terminal region of Tau (aa. 243-441).

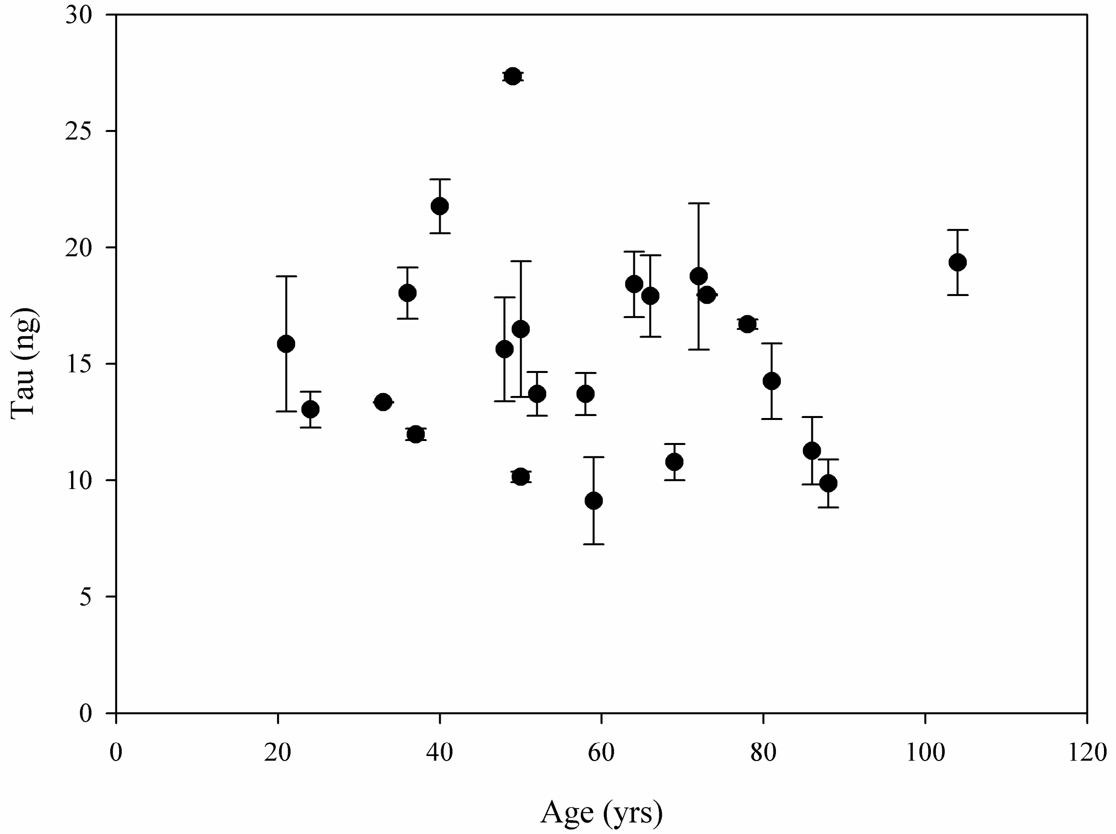

Supplementary Figure S3 Estimation of Tau in the soluble fractions of the PFC. Soluble PFC fractions of the cohort used in this study were diluted to 5 mg/mL using concentrations determined by the BCA assay of the brain homogenates. Triplicate 2µL aliquots of each sample were applied onto a nitrocellulose membrane. In triplicate 100, 50, 25, 12.5 and 6.25 ng of the commercial Tau standard were separately dotted onto the nitrocellulose. Both standards and samples were probed with the Dako Tau antibody (1:10000). A standard curve was generated from the Tau standards based on their intensity. The amount of Tau in each sample was estimated from the standard curve. Error bar +/-SD.
